# Enhancing glioblastoma treatment through the integration of tumor-treating fields

**DOI:** 10.3389/fonc.2023.1274587

**Published:** 2023-10-17

**Authors:** Katarzyna Szklener, Mateusz Bilski, Karolina Nieoczym, Dominika Mańdziuk, Sławomir Mańdziuk

**Affiliations:** ^1^ Department of Clinical Oncology and Chemotherapy, Medical University of Lublin, Lublin, Poland; ^2^ Department of Radiotherapy, Medical University of Lublin, Lublin, Poland; ^3^ Student Scientific Association at the Department of Clinical Oncology and Chemotherapy, Medical University of Lublin, Lublin, Poland

**Keywords:** glioblastoma, TTF, tumor treating fields, GBM, cancer treatment

## Abstract

Glioblastoma (GBM) represents a significant therapeutic challenge due to its aggressive nature. Tumor Treating Fields (TTFields) present a promising approach to GBM therapy. The primary mechanism of TTFields, an antimitotic effect, alongside numerous indirect effects including increased cell membrane permeability, signifies their potential in combination with other treatment modalities. Current combinations often include chemotherapy, particularly with temozolomide (TMZ), however, emerging data suggests potential synergy with targeted therapies, radiotherapy, and immunotherapy as well. TTFields display minimal side effects, predominantly skin-related, posing no significant barrier to combined therapies. The effectiveness of TTFields in GBM treatment has been demonstrated through several post-registration studies, advocating for continued research to optimize overall survival (OS) and progression-free survival (PFS) in patients, as opposed to focusing solely on quality of life.

## 1 Introduction

GBM, particularly its high-grade form traditionally referred to as Glioblastoma multiforme (GBM), is an extremely aggressive brain tumor that constitutes the majority of primary brain tumors in adults. Despite current treatment methods, the prognosis for GBM remains poor. However, a promising, relatively new therapy known as TTFields has emerged and received approvals for supratentorial GBM treatment and IDH mutant WHO G4 astrocytoma (1, 2).

TTFields are a non-invasive cancer therapy that utilizes alternating electrical fields to specifically target rapidly dividing tumor cells (2). The TTFields Optune device has been approved in the United States since 2011 as a treatment for adults (over 22 years old) with histologically-confirmed GBM. In 2015 the FDA approved TTFields for the treatment of adult patients with newly-diagnosed GBM (3). It is accepted both a monotherapy for recurrent GBM and in combination with temozolomide for newly diagnosed GBM following maximal debulking surgery and completion of radiation therapy or concurrent radiochemotherapy with temozolamide. TTFields serve as an alternative when standard medical therapy options have been exhausted (3, 4). Consequently, TTFields have become integrated into the standard-of-care treatment for GBM (3). The aim of this paper is to provide an overview of the current understanding of TTFields’ mechanisms of action and their application in the treatment of GBM and other cancers, as well as the ongoing clinical trials in these areas.

The purpose of this paper is to establish the current state of knowledge on the effectiveness of the use of TTFields in the treatment of recurrent and newly diagnosed GBM in monotherapy and combination therapy, as well as to present the current directions of clinical research on this topic.

## 2 TTFields’ mechanisms of action

### 2.1 Anti-mitotic effects

An electric field is a region around a charged particle or object within which a force would be exerted on other charged particles or objects. It is a vector field, with both magnitude (strength) and direction (5). Alternating electric fields are electric fields that change direction and magnitude in a periodic manner (6). In a broader context, this phenomenon can impede spatial configurations such as cellular divisions due to the inherent challenges faced by organelles in facilitating the movement of charged molecules (7). For instance, it may inhibit microbial growth (8). Therefore, the basic biological mechanism intended for the operation of the fields applied in clinical oncology is to stop the division of cancer cells (9). Although the pre-clinical and clinical data are promising, the mechanisms of TTFields are not fully elucidated (10).

The disruption of septin-microtubule complex formation is identified as a strong contributing factor to several outcomes, including aneuploidy (an abnormal number of chromosomes in a cell), impaired cleavage furrow function (a crucial step in cell division), and increased cell death in cancer cells. Therefore, the disruption of this complex formation leads to cell deaths through these mechanisms (10, 11).

TTFields exert their effects on mitosis-related processes, specifically targeting DNA replication and the cell cycle, particularly during the anaphase of mitosis, because they cause abnormal chromosomal segregation, altering the duration of mitotic phases, and reducing cellular proliferation (10–13), although TTField antimitotic activity is evidence to impact telophase, and cytokinesis as well (13). TTFields also affects the cell cycle by inducing nuclear envelope disruption and micronucleus formation, particularly in the S phase (11).

Generally, the impact of TTFields on the progression of the cell cycle depends on its specific phase, wherein entry into the G1/S phase is essential for TTFields to effectively inhibit tumors. TTFields can induce diverse cell cycle arrests, with G2/M arrest commonly observed in glioma cells. The occurrence and timing of the Sub G1 peak, indicating apoptosis and the presence of specificsized DNA fragments, vary among different cell lines (10, 11).

Additionally, in certain studies, TTFields have been linked to necrosis, immunogenic death, and necroptosis. The mechanisms underlying cell death induced by TTFields involve P53-dependent pathways, elevation of reactive oxygen species, caspase-dependent/independent pathways, and MGMT-independent pathways (10, 11).

Additionally, TTFields can impact the expression of chromosomal maintenance genes, further interfering with normal cell cycle progression (10). It is also suspected that TTFields can disrupt DNA repair mechanisms in cancer cells. This is characterized by increased levels of residual γH2AX, a marker of DNA damage, and decreased expression of ATM, an early activation factor in DNA repair (10).

TTFields is proven to autophagy in gliomas or GBM through abnormal mitosis and other factors (11).

The mechanisms of autophagy associated with the action of TTFields are mostly associated with cellular stress (10–12, 14, 15) or with the activation of programmed cell death pathways (14). RNA analysis revealed a >2 fold increase in autophagy-related genes in TTField-treated cells. Observable cellular changes consistent with autophagy included heightened presence of vacuoles, autophagosomes, and alterations in mitochondria and endoplasmic reticulum. TTFields induce autophagy by causing replication stress, resulting in reduced mitotically active cells and increased expression of autophagy markers (11, 12).

Mechanistically, TTFields upregulate AMPK and facilitate the interaction of Beclin1 with Vps34 or Atg14L while inhibiting its interaction with Bcl2. TTFields-induced miR-29b inhibits AKT2 expression (11, 12). However, autophagy may also serve as a protective mechanism for tumor cells against TTFields. Inhibition of AMPK or ATG7 can suppress TTFields-induced autophagy and result in cell death, indicating that TTFields-induced autophagy depends on AMPK activation (11). The role of autophagy in TTFields’ cytotoxic effects or resistance mechanisms remains unclear (12).

### 2.2 Membrane-related effects

Further mechanisms of the intracellular interaction of TTFields include, in particular, interference with cell membrane proteins or other factors destructive for membranes as caspase-mediated pyroptosis and membrane disruption (11). Most researchers believe that cell membrane damage is a separate mechanism that promotes cell destruction and non-proliferation as an additional cellular stress factor (10, 11). Membrane-related effects apply not only to the cell membrane, but in particular to the nuclear membrane (11).

When high-pulsed electric fields are applied to cells, they may directly or indirectly increase membrane permeability. This mechanism is important because it is observed only in cancer cells and not in healthy cells (15). As TTFields also increase tumor cell membrane permeability without affecting normal cell membranes, demonstrating the selective action of TTFields on cancer cells, shorter treatment durations have minimal effects on normal cells (11).

In turn, the pharmacodynamic derivative effects of the use of TTFields are associated with the loss of cell membrane tightness (10, 14, 15). They allow transmembrane transport of substances up to a certain size, affecting ion channels such as CACNA1C, influencing cell cycle progression and migration, altering cell membrane potential and intracellular ion homeostasis, and disrupting tight junction proteins of the blood-brain barrier, temporarily increasing its permeability and expanding the potential for intracranial drug applications (15, 16). TTFields exposure results in reversible changes in membrane permeability, with the cell membrane integrity being repairable (11). Greater membrane permeability makes TTFields particularly suitable for use in combination therapy, especially with chemotherapy (16).

Moreover, TTFields exhibit antiangiogenic effects by inhibiting tube formation and downregulating angiogenesis-related molecules (11, 14). Therefore, their effect on cell membranes may also cause larger effects, at least on the scale of the surrounding tissues. These effects of TTFields have important implications for reducing recurrence, enhancing drug delivery, and inhibiting tumor growth and progression (14).

A derived antitumor mechanism of TTFields may be the prevention of tumor infiltration. TTFields inhibit cell migration and invasion by dysregulating cytoskeletal structures and proteins related to the epithelial-mesenchymal transition (14). Thus, TTFields seem to universally inhibit the metastatic spread of tumors by hindering cancer cell motility, suppressing ciliogenesis, affecting EMT-related biomarkers, inhibiting ECM degradation, reducing angiogenesis, and inhibiting PI3K/AKT and MAPK signaling pathways. Ultimately, this leads to reduced migration, invasion, and aggressive properties of cancer cells (14, 15, 17).

### 2.3 Immunomodulative effects

TTFields treatment has been shown to activate systemic anti-tumor immune responses. These responses involve the release of damage-related molecular patterns, increased infiltration of antigen-presenting cells, promotion of immunogenic cell death, activation of macrophages, and pro-inflammatory cytokine secretion (10–20).

Immunomodulation is a relatively poorly understood mechanism of action of TTFields, although some studies have shown its high potential to enhance immune responses in cancer treatment (10, 11, 15). While in cancer cells TTFields exposure downregulates the MAPK and NF-κB pathways, an opposite effect is observed in mouse macrophages, where TTFields increases the phos-phorylation of IκBα and NF-κB p65, triggering the release of phosphorylated p65. The released p65 can then translocate into the nucleus, activating genes that are crucial for macrophage activation and the immune response. This mechanism promotes immunogenic cell death, stimulating the activation and recruitment of macrophages and other immune cells, thus enhancing the recognition and clearance of cancer cells (10, 15).

In mouse models of lung and colon cancer, the application of TTFields activated innate and adaptive immunity and increased leukocyte recruitment. Importantly, it was observed that TTFields did not significantly alter the antitumor activity of activated T cells or the functionality and viability of CAR T-cells, implying that TTFields could enhance immune responses while preserving the functionality of immune cells participating in antitumor activities (10, 20).

TTFields probably lead to the upregulation of proinflammatory cytokines such as IL-1β and TNFα, which play a role in macrophage activation and recruitment (10). Additionally, TTFields modulate gene expression in GBM (GBM) samples, upregulating antitumor genes and downregulating protumor genes. TTFields also induce the expression of immunogenic markers on cancer cells, including calreticulin (CRT) on the cell surface and the release of high mobility group box 1 protein (HMGB1), indicating immunogenic cell death. These observations suggest that TTFields can foster an inflammatory and immunogenic environment that aids immune cell recruitment and enhances antitumor immune responses (10, 18).

While TTFields inhibits the proliferation of immune cells such as RAW264.7 macrophages and T cells, it also maintains their functional activation status. This is evidenced by changes in morphology, molecular changes, and the secretion of factors like reactive oxygen species, nitric oxide, IL-1β, TNF-α, and IFNγ. TTFields can also induce immunogenic death of tumor cells, leading to the release of danger signals such as HMGB1, ATP, and calreticulin, which activate dendritic cells and promote CD45+ leukocyte enrichment. Furthermore, TTFields can enhance immune cell infiltration, including CD8 T cells, in tumor tissues (10, 18).

The effects of TTFields on immune responses have been associated with the TP53 and RhoA signaling pathways. TTFields-induced abnormal micronuclear clusters can activate the cGAS-STING pathway or the AIM2/caspase1 inflammasome release, leading to the production of proinflammatory cytokines and type I interferon, which contribute to adaptive immune activation. TTFields may also upregulate immune checkpoints and other molecules associated with immune activation, providing a potential foundation for combination therapy with immunotherapy. However, further investigation is needed to fully understand the molecular mechanisms underlying these effects of TTFields on immune cells (11, 14, 15).

In addition to downregulating the MAPK and NF-κB pathways in cancer cells, TTFields exposure was found to have an opposite effect on mouse macrophages, increasing phosphorylation of IκBα and NF-κB p65. This led to the release of phosphorylated p65, allowing it to translocate into the nucleus and activate genes necessary for macrophage activation and immune response. This phenomenon promotes immunogenic cell death and the activation and recruitment of macrophages and other immune cells to enhance cancer recognition and clearance (10).

TTFields exposure can enhance tumor cell clearance through macrophage activation and immune cell recruitment. In lung and colon cancer mouse models, TTFields increased leukocyte recruitment and activated innate and adaptive immunity. TTFields did not significantly affect the anti-tumor activity of activated T cells or the functionality and viability of CAR T-cells. These findings suggest that TTFields may enhance immune responses while maintaining the functionality of immune cells involved in antitumor activities (10).

A study by Barsheshet Y. et al. showed that TTFields treatment of *in vitro* M1 and M2 macrophages has also resulted in increased production of proinflammatory cytokines (CXCL1, IL18, IL23, IL12p70, TNFα, IL12p40, CCL22, G-CSF, CCL17, and IL1β) as well as skewing of M2 macrophages to M1 phenotype. That observation suggests that TTFields may modulate the immune response in a way that promotes inflammation and potentially promote adaptive immune response against tumor neoantigens. TTFields also modulate gene expression in GBM samples, upregulating antitumor genes and downregulating protumor genes (10, 14, 15, 18, 19).

Furthermore, TTFields induce the expression of immunogenic markers on cancer cells, including calreticulin (CRT) on the cell surface and the release of high mobility group box 1 protein (HMGB1), indicating immunogenic cell death. These findings suggest that TTFields can promote an inflammatory and immunogenic environment that facilitates immune cell recruitment and enhances antitumor immune responses (10, 11).

While TTFields inhibit the proliferation of immune cells such as RAW264.7 macrophages and T cells, it also maintains their functional activation status, as evidenced by morphological changes, molecular changes, and secretion of factors like reactive oxygen species, nitric oxide, IL-1β, TNF-α, and IFNγ. TTFields can induce immunogenic death of tumor cells, leading to the release of danger signals such as HMGB1, ATP, and calreticulin, which activate dendritic cells and promote CD45+ leukocyte enrichment (11). TTFields can also increase immune cell infiltration, including CD8 T cells, in tumor tissues. TP53 and RhoA signaling have been implicated as potential targets of TTFields in modulating immune responses (11).

TTFields-induced abnormal micronuclear clusters can activate the cGAS-STING pathway or the AIM2/caspase1 inflammasome release, leading to the production of proinflammatory cytokines and type I interferon, which contribute to adaptive immune activation. TTFields may upregulate immune checkpoints and other molecules associated with immune activation, providing a basis for combination therapy with immunotherapy. However, the molecular mechanisms underlying these effects of TTFields on immune cells require further investigation (10, 11).

TTFields treatment activates systemic anti-tumor immune responses, involving the release of damage-related molecular patterns, increased infiltration of antigen-presenting cells, promotion of immunogenic cell death, activation of macrophages and pro-inflammatory cytokine secretion, disruption of the nuclear envelope leading to the activation of DNA sensors and inflammasome, and subsequent upregulation of pro-inflammatory cytokines and interferons, thereby creating an intrinsic immune platform against cancer (15).

### 2.4 Other effects

Other mechanisms of action of TTFIields are less well understood. Study by Patel et al. study found TTFields significantly reducing the abnormal glycolysis in human GBM (a type of brain cancer), by lowering the expression of the biomarker pyruvate kinase M2 (PKM2), and this reduction can be non-invasively detected using a novel radiotracer, [18F]DASA-23 (20). Perhaps, this research suggests a novel approach to therapy. The identification of pyruvate kinase M2 (PKM2) as a biomarker for GBM response to TTFields could help clinicians to monitor the effectiveness of this treatment in real-time. This might allow them to make more informed decisions about whether to continue, adjust, or stop the TTFields therapy (20). However, it is important to note the need for further targeted research to determine how changes in PKM2 relate to the efficacy of tumour field treatment in GBM.

In addition to the aforementioned factors, it is likely that TTFields produce systemic physiological effects, thereby establishing connections with previously existing system modalities. Genilal et al. found that TTFields used for GBM treatment can lead to localized temperature increases in the scalp and brain, with maximum temperatures reaching approximately 41.5°C and 38°C, respectively. These temperature changes may impact blood-brain barrier permeability and blood flow, emphasizing the importance of optimizing TTFields delivery and considering potential thermal effects to ensure uninterrupted treatment administration. Their study highlights the need to adjust new current injection mode to minimize thermal impacts (21).

Currently, there are several ongoing trials (NCT03405792, NCT04221503, NCT03477110, NCT04474353, NCT04469075, NCT03705351, NCT04397679) labeled as investigating the molecular mechanisms of TTFields in the treatment of GBM. However, none of these studies explicitly focus on this particular issue, as neither the descriptions nor objectives indicate its central importance.

### 2.5 Skull remodeling

TTFields are delivered through transducer arrays that are placed on the scalp. Because the electric fields are applied externally, the shape, thickness, and density of the skull can affect the distribution and intensity of the fields that reach the tumor site (3, 6, 22–25).

Skull remodeling, a process of bone deposition and resorption that maintains bone health, can have a significant impact on the treatment outcomes of TTFields therapy. Changes in skull shape and density due to remodeling may affect the depth and intensity of TTFields penetration and thus the therapeutic effect on the tumor. Precise fitting of the transducer arrays and the consequent efficient delivery of TTFields may also be impacted by skull remodeling (26). Therefore, skull remodeelling such as minor craniectomy or distributed burr holes, can enhance the electrical field strength in TTFields therapy by up to ~100%, potentially improving clinical outcomes for patients (22).

In some studies, combining skull remodeling surgery with TTFields treatment has shown promise in patients with recurrent GBMs, improving OS and PFS. The feasibility and safety of this approach were demonstrated in a phase 1 trial, leading to the initiation of a phase 2 trial. Additionally, combining hyperthermia with TTFields has been shown to enhance the therapeutic effects on GBM cells, resulting in inhibition of cell migration, increased apoptosis, and downregulation of STAT3 expression levels (17).

These procedures create approximately 15 mm diameter burr holes, which can channel electricity through the path of least resistance, increasing electric current in the tumor. A phase 1 clinical trial with 15 participants demonstrated safety and promising results in terms of increased OS (22).

Further research seems needed to fully elucidate the implications of skull remodeling on the effectiveness of TTFields therapy and to develop strategies for addressing these challenges in the clinical setting.

The ongoing OptimalTTF-2 trial is investigating the combination of skull remodeling surgery, TTF, and best practice medical oncological therapy for first recurrence in GBM patients. The phase 2 trial aims to validate the efficacy of this intervention, with primary endpoints including overall survival and secondary endpoints such as progression-free survival, objective tumor response rate, quality of life, and toxicity. This treatment modality has the potential to significantly increase overall survival in patients with first recurrence glioblastoma (27, 28).

### 2.6 Dosimetry and treatment planning

The TTFields treatment planning process includes clinical evaluation and creation of a patient-specific computational model, optimization of the transducer array layout, and definition of a treatment plan (29).

Optune® Therapy (TTFields) uses sequential activation of two pairs of electrodes, resulting in two consecutive fields active for 1 second each. Previous studies focused on individual field distributions but did not consider spatial field correlation, leading to variations in anticancer effect based on cell division direction. A method to estimate uncorrelated field components within tissue and within one activation cycle has been proposed, accounting for average field strength and direction-al efficiency. Calculations utilize finite element methods to obtain field distributions (22, 30).

The patient must be instructed on how to place the transducer array and further imaging is performed to assess disease progression. The three key elements of successful TTFields treatment planning are accurate TTFields dosimetry, creation of patient-specific models, and advanced imaging to monitor response to therapy (24).

Patients who received higher doses into the tumor bed showed better treatment results in the ET-14 study (24, 31–33).

The average field strength, power loss density and dose density in the tumor bed were calculated and values were identified that divided the patients into two groups with the most significant difference in OS. Patients receiving a mean dose of TTFields ≥ 0.77 mW/cm3 had longer median PFS (8.5 vs 6.7 months) and OS (25.2 vs 20.4 months) compared to patients receiving a lower dose. Similarly, higher TTFields in the tumor bed (≥ 1.06 V/cm) were associated with longer median PFS (8.1 vs 7.9 months) and OS (24.3 vs 21.6 months). Higher doses were also correlated with improved quality of life. These findings suggest that optimizing TTFields dosimetry and treatment planning using simulation models has the potential to improve patient outcomes (14).

Other treatment in action may influence the doze, although seems yet uncertain. Combining adjuvant radiochemotherapy with simultaneous tumor treatment fields (TTFields) was found to be feasible and did not result in clinically significant dose deviations in patients with glioblastoma multiforme, according to a dosimetry study by Guberina et al. (34). Stachelek et al. conducted a study simulating radiation plans with TTFields on a skull model and found that the placement of TTFields arrays did not affect the target volume coverage of radiotherapy (27).

Various numerical methods can be used in dosimetry studies, including the finite element method (26). Increasing the intensity of TTFields in the tumor bed improved patient outcomes in GBM, according to Urman et al. study utilizing realistic head models created from MRI scans and finite element simulations (30).

Also personalized therapeutic decision making, including the use of TTFields and/or potentially active NGS-based targeted or immunotherapeutic agents of patients’ tumors, are now routine in a neuro-oncology clinic (33).

## 3 TTFields in GBM Treatment

### 3.1 Monotherapy

Data from the EF-14 trial led to the approval of TTFields by the FDA in 2015 for newly diagnosed GBM. It is worth emphasising that EF-14 was the first study since the STUPP 2005 NEJM TMZ study to introduce another treatment option as part of the newly diagnosed treatment regimen.

TTFields is indicated as a treatment for adult patients (22 years of age or older) with histologically-confirmed glioblastoma multiforme. Combined with temozolomide is indicated for the treatment of adult patients with newly diagnosed, supratentorial glioblastoma following maximal debulking surgery and completion of radiation therapy together with concomitant standard of care chemotherapy (1, 22, 23, 35–47).

Adding TTFields therapy to standard of care treatment was significantly associated with improved survival for patients with newly diagnosed GBM. Additionally, the magnitude of the survival benefit with TTFields in the real-world setting was shown to be consistent with that of the pivotal EF-14 trial, with an increase in median OS of approximately 5 months, and an overall reduction in risk of death in the 30–40% range versus standard chemoradiotherapy alone (37).

In a phase III trial conducted on patients with recurrent GBM, the effectiveness of TTFields therapy was evaluated in comparison to active chemotherapy, and TTFields monotherapy exhibited minimal toxicity, improved quality of life, and demonstrated comparable efficacy to chemotherapy (38), although some publication indicate limited benefits only (37).

Although tumor treating fields have shown promise, therapeutic benefit does not extend to all patients. Therefore, research identifying potential biomarkers predicting TTFs effectives is of utmost importance. In Dono et al. Study, among 149 patients with recurrent GBM (rGBM), 29 (19%) were treated with TTFields, and no significant difference in median PPS was observed between patients who received TTFields and those who did not. However, within the TTFields-treated group, patients with PTEN-mutant rGBM had improved PPS compared to PTEN-WT rGBM patients, and within the PTEN-mutant subgroup, those treated with TTFields had longer median PPS compared to those who did not (while no PPS benefit was observed in PTEN-WT patients receiving TTFields). It may suggest that TTFields therapy provides a significant PPS benefit specifically in PTEN-mutant rGBM (40).

Although TTFields monotherapy failed to demonstrate a superior OS compared to best physician’s choice chemotherapy, improved response rate (14% vs 9.6%) with a comparable OS (HR 0.86 [CI 0.66–1.12]; p = 0.27) suggested clinical activity (39).

In post-registration trials comparing TTFields to best supportive care (BSC) from study EF-11, the median OS was 7.4 months vs. 6.4 months (p = 0.053), indicating a trend towards improved survival with TTFields. The hazard ratio (HR) for OS was 0.64 (95% CI 0.46-0.91, p = 0.012), showing a significant advantage in survival for the TTFields group. In the per-protocol population, the median OS was 8.1 months with TTFields versus 6.5 months with EF-11 BSC (p = 0.045), further supporting the benefit of TTFields. The incidence of adverse events (AE) was lower with TTFields (67%) compared to EF-11 BSC (95%), and the median time to treatment failure was longer in the TTFields arm (3.3 months) versus the BSC arm (1.6 months) (HR = 0.53, 95% CI 0.41-0.68, p < 0.0001) (43).

A newer post-registration study showed median OS comparable between TTFields monotherapy and standard of care (7.4 months vs. 6.4 months), with the hazard ratio (HR) for TTFields monotherapy at 0.66 (95% CI: 0.47-0.92), in the intent-to-treat population, indicating a lower risk of death compared to standard care (p=0.016). While in per-protocol population, median OS was significantly longer for TTFields monotherapy compared to standard of care (8.1 months vs. 6.4 months). The HR for TTFields monotherapy was also better at 0.60 (95% CI: 0.42-0.85; p=0.004). Extended use of TTFields therapy (≥18 hours/day averaged over 28 days) demonstrated increased efficacy (43).

More extensive data on the use of TTFields as the solo treatment in patients with newly diagnosed GBM are still lacking. Although there is little evidence that exposure to TTFields could be superior to traditional treatment. As temozolomide chemotherapy is continued after resection, at least to progression, the results presented so far relate to the use of TTFields in combination with chemotherapy (43).

Cell culture experiments confirmed that TTFields reduce the proliferation of different glioma cell lines in a field strength- and frequency-dependent manner. The reduction of the cell number depends on the duration, intensity, and frequency of the applied TTFields. Moreover, commutation time of the electrical fields seems to be an additional exponent of the effectiveness of this method. The power absorption in the mitotic furrow region is frequency-dependent. Variable cell diameters, and thus variabilities in the membrane capacitance as well as variability in the conductivity of the cytoplasmic cytosol should be taken into account. At low frequencies the cell membrane effectively shields the inner cell from the electric fields, due to the high resistance compared to the external culture medium and the cytosol. Meanwhile, at higher frequencies the membrane’s capacitance provides a parallel conducting path for displacement currents, which increase with frequency and begin to shorten the membrane’s resistance at around 1 MHz (44). In the study conducted by Berkelmann L. et al. it was stated that TTFields application at frequencies in the range of 100 kHz to 200 kHz significantly reduced the cell proliferation, while higher frequencies did not (44). Salzberg et al. treated a single patient with newly diagnosed GBM using TTFields (100–200kHz; field intensity:0.7 V/cm) for 128 days (22).

Currently, no reviled clinical trial is testing the efficacy or safety of TTFields monotherapy in GBM, but a large-scale quality-of-life study, NCT04717739, may include patients with relapsed GBM treated with TTFields alone.

### 3.2 TTFields combined with chemotherapy

TTField is mostly used with chemotherapy. This applies in particular to TMZ treatment, which is initiated in conjunction with resection and continued after it (1, 22, 23, 35–47).

Chemoresistance remains a significant challenge in GBM (23) and glioma (31) therapy. Experimental data suggests that the resistance of GBM to TMZ may involve more complex mechanisms than initially anticipated, necessitating consideration of multiple factors to understand the high rates of relapse (23).

The implementation of exposure to TTFields during TMZ chemotherapy is considered one of the main ways to overcome GBM chemoresistance (11, 12, 17, 23, 31, 32).

Combining TTFields, particularly with TMZ, has shown promise in overcoming chemoresistance and improving treatment outcomes in GBM (11, 17).

Combining TTFields with maintenance temozolomide (TMZ) in newly diagnosed GBM patients increased OS and PFS. Simulation-based studies showed higher dose density of TTFields correlated with better survival outcomes. Ongoing trials are investigating the synergistic effects of TTFields with radiation therapy and TMZ. These findings suggest TTFields as a promising treatment option for GBM (48).

In a study by Toms et al., the EF-14 *post hoc* trial included 450 patients with newly diagnosed GBM. The treatment group received involved TTFields combined with TMZ, resulting in a median OS of 20.9 months and a median PFS of 6.7 months. In a group of 229 ndGBM patients who received TMZ alone, the median OS was 16.0 months, and the median PFS was 4.0 months. Among patients using TTFields for more than 21.6 hours per day, the median OS was 24.9 months, and the median PFS was 8.2 months. Similarly, patients using TTFields for 19.2-21.6 hours per day had a median OS of 21.5 months and a median PFS of 8.1 months (32).

For patients using TTFields for 16.8-19.2, 14.4-16.8, 12-14.4, and 7.2-12 hours per day, the median OS and median PFS were as follows: 21.7 months and 7.7 months, 19.9 months and 5.4 months, 18.0 months and 4.2 months, and 17.9 months and 4.8 months, respectively. Finally, patients using TTFields for less than 7.2 hours per day had a median OS of 18.2 months and a median PFS of 5.9 months (32).

Retrospective research on Japanese patients shows that the survival rates achieved with TTFields therapy are comparable to or slightly higher than BSC (reported in EF-14 trial). TTFields 1-year OS rate was 77.9% (95% CI 60.6–88.3) and the 2-year OS rate was 53.6% (95% CI 35.5–68.7), while in EF-14 the 1-year OS rate reported in the EF-14 trial was 73% (CI: 69–77%) and the 2-year OS rate was 43% (CI: 39–48%). The 6-month PFS rate was 77.5% (95% CI 61.2–87.6) in compare to 56% (CI: 51–61%) in EF-14. It may suggest slightly higher efficacy of TTFields in compare to BSC (32).

Preclinical and clinical studies conducted on various solid tumours have demonstrated additive or synergistic effects when TTFields are combined with various chemotherapeutic agents, including pemetrexed, cisplatin, paclitaxel, erlotinib, and 5-FU (11, 17).

Additionally, combining TTFields with other chemotherapeutic agents, such as lomustine has demonstrated clinical efficacy and potential benefits, particularly in patients with specific genetic characteristics like O6-methylguanine DNA methyltransferase (MGMT) promoter methylation (17).

Moreover, based on a preclinical study, the combination of Withaferin A and Tumour Treating Fields resulted in blocking the proliferation of actively growing, human-derived glioma cells. Therefore, their synergistic effect represents promising approach to treat GBM (49).

AEs (Adverse Events) commonly associated with TTFields treatment are primarily related to dermatological reactions. These reactions may include irritant or allergic contact dermatitis, ulcers, and cutaneous infections. These AEs are mainly caused by the sensors applied to the scalp during treatment. There are five types of dermatological AEs commonly observed with TTFields treatment: hyperhidrosis (excessive sweating), pruritus (itching), contact dermatitis, skin ulcers, and skin and soft tissue infections. Managing these AEs involves implementing prophylactic measures such as using topical corticosteroids and antibiotics, ensuring a dry scalp, practicing careful shaving techniques, and regularly repositioning the arrays to minimize skin-related complicat ions (15, 17, 48, 50–53).

Castillo-Vaca et al., conducted a comprehensive literature search in PUBMED/MEDLINE identifying 37 articles matching criteria of “glioblastoma” and “temozolomide” occurrence. Their results showed that the use of TTFields in combination with TMZ increased both PFS and OS compared to standard of care. Other therapies such as ipilimumab plus temozolomide and veliparib did not significantly improve OS or PFS rates compared to RT with TMZ (54).

One of the main challenges associated with the combination of TTFields and chemotherapy is determining the predictors of successful treatment. Methylation of the O6-methylguanine DNA methyltransferase promoter (MGMT) seems to be highly associated with improved response to TMZ chemotherapy and longer OS, but the occurrence of MGMT promoter methylation changes between primary tumor and relapse remains uncertain due to limited and contrasting data (55).

An early Phase 1 study (NCT03477110) is intended to test novel TTF device (NovoTTF-200A) in combination with RT and TMZ chemotherapy. This trial secondary objectives are to collect tumor O(6)-Methylguanine-DNA-methyltransferase (MGMT) methylation status and isocitrate dehydrogenase (IDH) mutation status, as well as to evaluate the level of circulating tumor deoxyribonucleic acid (DNA) in GBM patient serum during treatment. NCT04671459 is also intended to check the same mechanism but during TTFields + RT treatment.

Currently there are also two other clinical trials involving chemotherapy combined with TTFields in GBM treatment (NCT04474353, NCT03705351).

NCT04474353 is intended to determine the safety of TTFields started concurrently with 5 fraction stereotactic radiosurgery (SRS) and temozolomide for newly diagnosed GBM, with a secondary objective to determine efficacy for the combination of TTFields started concurrently with 5.

### 3.3 TTFields combined with RT

Both TTField and RT act by slowing down DNA damage repair (32). Combining TTFields with initial RT and TMZ therapy in newly diagnosed GBM showed feasibility and safety, with a median PFS of 8.9 months and low-severity local dermatological complications in 80% of patients (32, 53).

TTFields therapy combined with RT has shown synergistic effects in enhancing the efficacy of radiation in glioma cells. Preclinical studies have demonstrated that this combination treatment prevents cell migration, promotes apoptosis, and mitotic abnormalities. Clinical studies have reported promising results, including improved PFS and complete radiological responses in patients with newly diagnosed GBM receiving TTFields in combination with RT and TMZ chemotherapy. Furthermore, the combination treatment has been well-tolerated, with good skin toxicity profiles and higher PFS compared to historical controls (17).

RT and TTFields have a combined effect regardless of the sequence of administration. Higher radiation doses and proton therapy are more effective when combined with TTFields (11).

Stein et al. reported a case of complete radiological response in thalamic GBM after treatment with proton therapy, TMZ, and TTFields (29).

In combination with RT, TTFields treatment may increase skin dose, but studies suggest that the clinical significance of this increase is minimal. Nevertheless, scalp doses should be closely monitored during treatment planning. Skin toxicity remains a common challenge in TTFields therapy and other glioma treatments such as RT and antiangiogenic therapy (48).

Currently two trialas including TTFields and RT treatment in GBM are ongoing (NCT04474353, NCT04671459). NCT04671459 is aimed to establish if the combination of SRS and TTFields will complement each other, leading to improved outcomes with minimal toxicity. The study is an open-label, phase II trial involving 40 participants with recurrent GBM, and the treatment will be initiated within a specific timeframe based on imaging evaluation. The study also aims to assess the MGMT gene promoter methylation and IDH1/IDH2 mutation status of the participants. Stratification factors for OS include volume of tumor, PET-based treatment, SVZ invasion, MGMT methylation status, time to first progression, and TTFields compliance.

### 3.4 TTFields combined with immunotherapy and targeted therapy

TTFields and immunotherapy individually have demonstrated notable efficacy in treating GBM, thereby instigating a surge of interest in the potential synergistic effects of combining these treatment modalities. Despite a current lack of published clinical trials, preliminary investigations and observations indicate a positive interplay between TTFields and the immune tumor microenvironment (TME), thus suggesting a promising trajectory for the application of combined therapy (17).

The potential to augment cancer immunity by concomitantly employing TTFields and immunotherapy is currently under rigorous investigation. Several ongoing clinical trials are dedicated to exploring this therapeutic blend, encompassing combinations of TTFields with peptide vaccines and immune checkpoint inhibitors. One such trial (NCT03223103) is scrutinizing the utility of combining a mutation-derived tumor antigen vaccine with TTFields in the maintenance phase of TMZ therapy. Other investigations include the combination of pembrolizumab and TMZ for newly diagnosed GBM patients (NCT03405792), as well as nivolumab and ipilimumab for recurrent GBM (NCT03430791).

The findings from these trials hold immense potential in shaping future therapeutic strategies. Insights derived will be critical for clinicians in tailoring effective treatment plans, and for researchers to enhance our comprehension of the tumor microenvironment. This could ultimately lead to a marked enhancement in patient outcomes and OS rates, which currently remain disappointingly low for GBM (32). Thus, the amalgamation of TTFields therapy and immunotherapy could constitute a paradigm shift in the treatment of GBMs.

TTFields therapy has shown promising results in the treatment of GBM when used in combination with targeted therapies such as bevacizumab. Clinical studies report improved OS and PFS in patients receiving TTFields along with bevacizumab-based chemotherapy compared to bevacizumab monotherapy. Furthermore, TTFields combined with other targeted drugs such as dabrafenib and sorafenib have demonstrated clinical and radiological responses in patients harboring specific genetic mutations. Moreover, an enhanced effect of TTFields on glioma cells has been observed following the inhibition of the spindle assembly checkpoint (17).

The synergistic effect of combining TTFields with targeted drugs such as bevacizumab has been the subject of multiple phase 2 clinical trials for both newly diagnosed and recurrent GBMs (NCT01894061, NCT02743078, NCT02663271, NCT03687034, and NCT02343549). The efficacy of combining bevacizumab with TTFields has been confirmed, demonstrating improved response and survival outcomes. Other targeted drugs like sorafenib and dabrafenib are also being assessed in combination with TTFields, yielding encouraging preliminary results in sensitizing GBM cells and achieving clinical responses (32, 46).

Research endeavors are advancing beyond individual drug combinations and are exploring concurrent therapies, such as the combination of bevacizumab, TTFields, and hypofractionated stereotactic irradiation. These ongoing clinical trials embody hope for the treatment of primary or recurrent GBM by investigating a variety of therapeutic regimens that combine targeted drugs with TTFields. These strategies, if successful, could significantly advance the management of GBM, offering patients more effective treatment options and potentially improving prognosis (32).

To same extent, also TMZ may be seen as targeted therapy, sometimes seen as the best targeted option in GBM, although phase III trials indicated a median overall survival of 18-20 months (56). At the same time, one should be aware that treating it as a targeted therapy may introduce some confusion, because it can be targeted not only in the sense of genetic expression, but also in a technical way. One of the option of targeting of TMZ may be using magnetic nanobeads (57).

## 4 Conclusions

GBM, although common among head cancers, remains a very serious therapeutic challenge. TTFields seem promising, but there is no evidence for their use in monotherapy. On the contrary, the mechanism of action involving not only an antimitotic effect, but - above all - a number of indirect effects, including an increase in cell membrane permeability, leads to the conclusion that TTFields should be used mainly in combination with other agents. The most common therapeutic modality in combination with TTFields is chemotherapy, most often using TMZ.

However, targeted therapies and immunotherapy and RT also seem to benefit from the implementation of TTFields.

Relatively weak side effects, mainly of a skin nature, do not stand in the way of combining therapies.

Considering that post-registration studies usually show an increase in the effectiveness of treatment with the use of TTFields, one should not give up trying to find solutions that seek to maximize OS and PFS in favor of research focused on the quality of life.

In particular, research is needed that will optimize the dose and modes of interaction (perhaps with skull remodeling), which can ultimately lead to a permanent improvement in the results obtained with TTFields.

Despite cases of significant improvement, it does not seem that TTFields in itself can be a panacea for GBM, but it may turn out to be a universal catalyst for new therapeutic solutions in the field of various systemic therapy methods.

## Author contributions

KS: Writing – original draft. MB: Writing – original draft. KN: Writing – original draft. DM: Writing – original draft. SM: Writing – original draft.
